# A Peculiar Presentation of Syphilis as a Mysterious Rash: A Dermatological Dilemma

**DOI:** 10.7759/cureus.45328

**Published:** 2023-09-15

**Authors:** Priscila Lopez, Ifeoma Kwentoh, Mario Valdez Imbert

**Affiliations:** 1 Internal Medicine, Harlem Hospital Center, New York, USA; 2 Medicine, Columbia University, New York, USA

**Keywords:** #cutaneous syphilis, #hiv-hcv co-infection, #hiv aids, #kaposi's sarcoma-associated herpesvirus (kshv), #sexually transmitted infection (sti), #monkey pox virus rash

## Abstract

A renowned poet in the ancient city of Verona by the name of Girolamo Fracastoro coined the term syphilis in 1530. The stigma and shame that embodied this affliction has been time immemorial and disabling for patients. The hypothesis of the spread from the warm tropics of west and central Africa to the Iberian Peninsula accompanied by the slave trade has been a tale for centuries. Malignant syphilis is a rare skin manifestation of Treponema pallidum infection and a variant of secondary syphilis. The rash is frequently associated with HIV-infected patients, often with low cluster differentiation 4 (CD4) cell count. The authors reported a unique case involving a 46-year-old woman who presented with a one-week history of skin eruptions at various stages. Subsequent laboratory tests revealed a strong positive result for Treponema pallidum and a positive Rapid Plasma Reagin (RPR) test with a titer of 1:16. She received doxycycline because she had a history of penicillin anaphylaxis in the past. She did well, with a remarkable improvement in symptoms - a positive outcome for this catastrophic stigmatizing, rare diagnosis.

## Introduction

Treponema pallidum is a bacterium that belongs to the Spirochetes phylum, Spirochaetales classification and order [1,2]. Syphilis can present in many different ways based on the stage of the disease (primary, secondary, latent, and tertiary). Early secondary syphilis can manifest between two weeks and up to six months of exposure [3,4]. When syphilis occurs concurrently with HIV, it may module the classic skin presentations. A nonpruritic maculopapular rash is usually seen in syphilis and involves the palms and soles [5]. However, cutaneous necrotic sores and rash with eschar can mimic other disease processes when accompanied by prodromal symptoms of fever, myalgias, and arthralgia. Malignant syphilis is a rare condition that was first described in 1859, and since then, only a handful of cases have been reported [6,7,8,9]. The authors reported a case of a middle-aged woman with AIDS noncompliant with her highly active antiretroviral therapy (HAART) and malnourished presenting with an unusual, peculiar rash. The initial differential diagnosis included monkeypox, psoriasis, allergic dermatitis, drug-induced rash, and Kaposi sarcoma [10,11,12]. Dermatology was consulted and later diagnosed with T. pallidum infection.

## Case presentation

**Figure 1:**
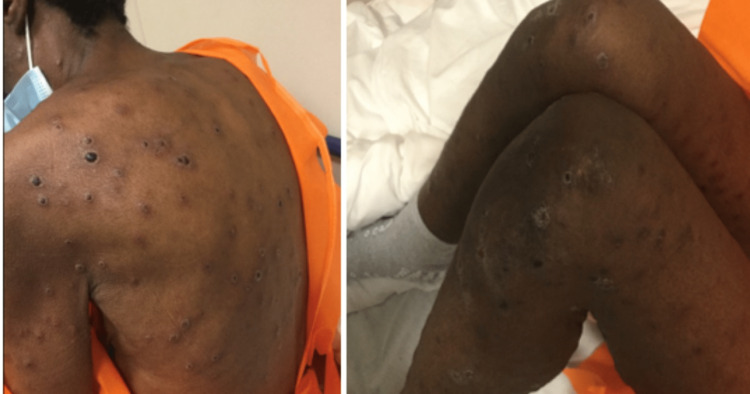
The patient had a generalized, nontender maculopapular rash characterized by erythematous papule-plaques, some with a necrotic center, ulcers covered with dark crusts, and the presence of blisters.

A 46-year-old woman with a past medical history of seizure disorder, asthma, gastroesophageal reflux disease (GERD), hypertension, HIV infection, schizoaffective disorder, depression, cocaine use disorder, and medication noncompliance presented with rash for one week, which was generalized, nonpainful but pruritic, and, according to her, started from the legs and disseminated to the whole body. There was no history of prodromal symptoms and no recent travel/sick contacts/use of new skin products. The last sexual encounter was approximately six months before admission, but she was not sure. She reported symptoms of fever, malaise, myalgias, hallucinations, and a depressed mood with suicidal ideation. Initially, the Department of Health (DOH) was contacted about the concern of monkeypox because an outbreak of this disease was happening at that time; however, DOH noted that monkeypox was unlikely due to the pattern. On presentation, the patient had stable vital signs with a blood pressure of 109/60 mmHg, a heart rate of 97 beats per minute, a respiratory rate of 16 breaths per minute, a temperature of 100.4 °C, and an oxygen saturation of 99% on room air. The physical examination revealed cachexia, with the oral mucosa showing no lesions or ulcers, and no signs of oral thrush. The skin exhibited a notable maculopapular rash, erythematous papule-plaques, some with necrotic centers, others with ulcers covered by dark crusts similar to eschars, as well as some blisters. These skin findings were present on the scalp, face, trunk, and all four extremities, but the palms and soles were spared (Figure 1).

Laboratory tests showed leukopenia 3.01 x 10^3 ^mcL^-1^, with lymphocytes at 10.5% and neutrophils at 82%. The cluster differentiation 4 (CD4) cell count was 60, and the viral load (VL) was 557. Fungitel results were negative. The Infectious Disease Specialty team was consulted and recommended a treatment plan involving vancomycin and levofloxacin. Additionally, Dermatology was consulted, and a biopsy was scheduled to investigate the possibility of a papulosquamous rash and possible psoriasis. The patient also received prophylaxis for Pneumocystis jiroveci pneumonia (PJP) with atovaquone 1.5 g daily. Blood cultures and urine cultures were negative. Chlamydia and gonorrhea tests came back negative. Tuberculosis was ruled out with three negative acid-fast bacilli tests and a negative quantiferon test. T. pallidum antibody tests were conducted, and the results returned positive. The RPR test was reactive with a titer of 1:16, and both the T. pallidum hemagglutination assay and enzyme immunoassay showed strong positive results (Table 1).

**Table 1:** Lab data. VL, viral load; AFB, acid-fast bacilli; RPR, Rapid Plasma Reagin; ABSO CD4, absolute cluster of differentiation cells 4

Lab data	Reference range and units	Patient data
Leukopenia	4.30 x 10^3^ to 11.00 x 10^3 ^mcL^-1^	3.01 x 10^3 ^mcL^-1^
ABSO CD4	489–1457 cells/mcL	60 cells/mcL
VL	<=20 copies/mL	229,000 copies/mL
Chlamydia amplification urine	Not detected	Not detected
Sputum AFB x3	Negative	Negative
Treponema pallidum Ab Screen I	Negative	Positive abnormal
RPR titer	<1:1	1:16
Treponema pallidum hemagglutination assay and enzyme immunoassay	Negative	Strongly positive
Quantiferon Plus TB	Negative	Negative

**Figure 2:**
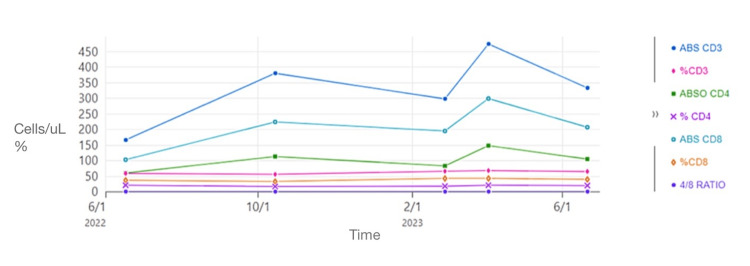
T-cell subset data. ABS CD3, absolute cluster of differentiation cells 3; ABSO CD4, absolute cluster of differentiation cells 4

Due to a previous history of an anaphylactic reaction to Penicillin, the patient was prescribed a 28-day course of doxycycline 100 mg for late latent syphilis. This decision was made because the time of exposure to syphilis was uncertain, and it was not a case of malignant syphilis. The patient’s rash eventually disappeared completely. Over time, the patient's VL and T-cell subset were monitored, and HAART treatment was resumed (Figure 2).

## Discussion

**Figure 3:**
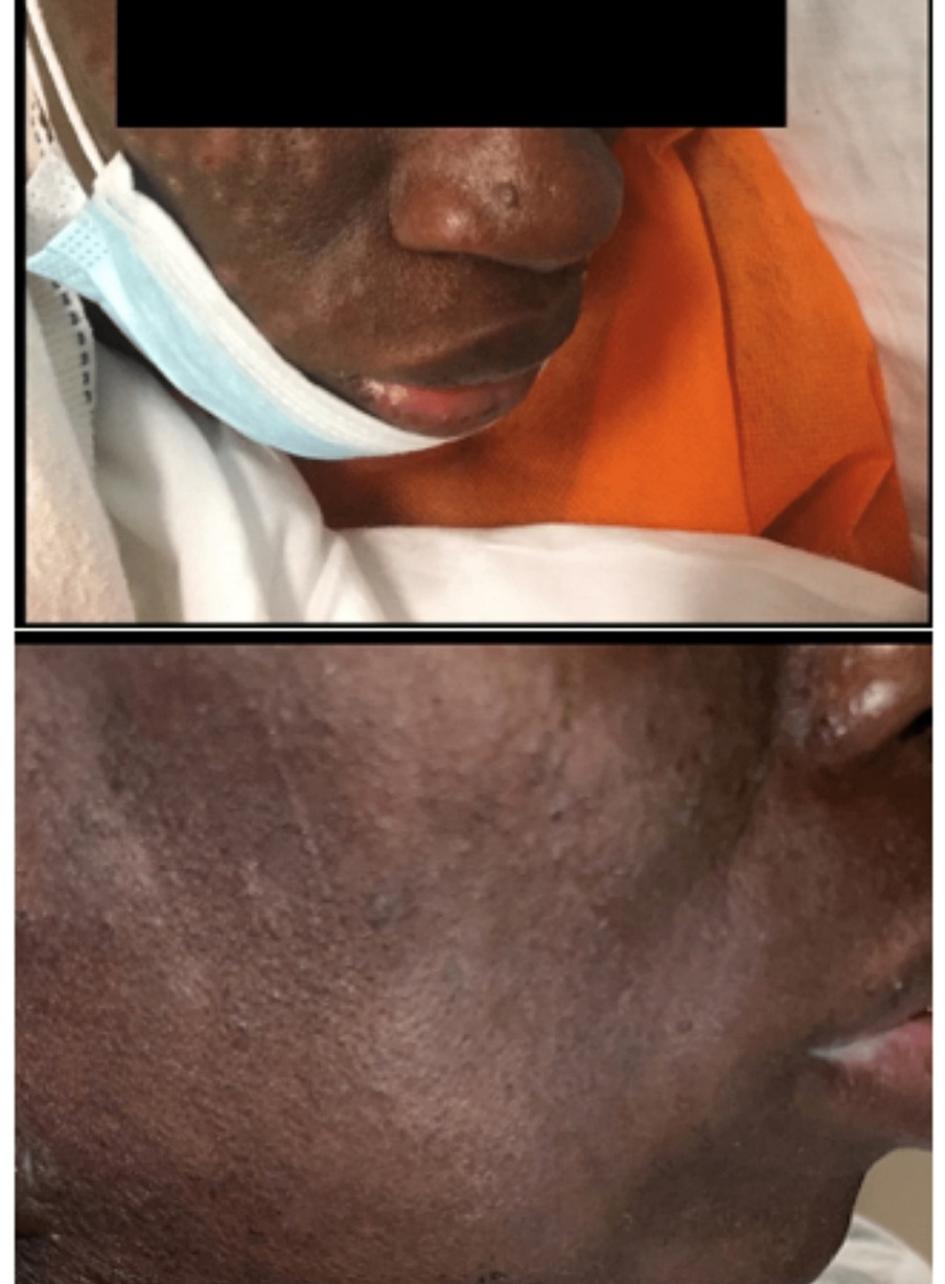
(Top panel) Before treatment; (bottom panel) dramatic improvement of lesions after treatment.

Syphilis is an infection caused by T. pallidum, and it can present with different symptoms, depending on the stage of the disease. Secondary syphilis is the most recognized manifestation of syphilis. Usually, the symptoms can appear after two to 12 weeks of exposure and up to six months [13,14]. It may initially present with a chancre, but the patient can remain asymptomatic until the disease progresses systemically [15]. The classic rash of secondary syphilis consists of painless, macular, reddish, or copper-colored lesions on the palms of the hands or soles of the feet but can be extremely variable as seen in our case with generalized necrotic papule-like lesions [15]. The rash can mimic other disease processes, including pityriasis rose, Rocky Mountain spotted fever, contact dermatitis, erythema multiforme, psoriasis, and drug eruptions. One could argue that the cutaneous eruption can be a result of a drug-induced rash or as seen in Jarisch-Herxheimer's reaction; however, a detailed medication history always comes in handy to eliminate such differentials. Malignant syphilis was first documented in 1859 and was evaluated as secondary vs. tertiary syphilis, but in 1897, it was concluded as a presentation of secondary syphilis [15]. Our case had a dramatic improvement after diagnosis and treatment was completed. The first-line treatment was penicillin, but in case of anaphylaxis, doxycycline was used [5] (Figure 3).

Monkeypox was a great contender for this case due to the outbreak that was happening during this patient presentation; however, it was a less likely diagnosis due to history and risk factors [16].

## Conclusions

In our presented case, the patient tested positive for the RPR test with a titer of 1:16. Additionally, she showed strong positive results for both the Treponema pallidum hemagglutination assay and enzyme immunoassay tests, which aligns with our working diagnosis of malignant syphilis. Due to the history of anaphylaxis with penicillin, the patient was treated with doxycycline 100 mg every 12 hours for 28 days, and the lesions improved remarkably within days following treatment. Physicians should maintain a high index of suspicion for this diagnosis when sudden generalized skin eruptions occur in immunocompromised patients.

## References

[1] Tampa M, Sarbu I, Matei C, Benea V, Georgescu S (2014). Brief history of syphilis. J Med Life.

[2] Avallone G, Cavallo F, Susca S (2022). Oral doxycycline in HIV-related synchronous malignant syphilis and condyloma lata. Ital J Dermatol Venerol.

[3] Li J-H, Guo H, Gao X-H (2015). Multiple skin ulcers from malignant syphilis. Lancet.

[4] Lueking R, Lazarte S (2022). Malignant syphilis. N Engl J Med.

[5] Kumar B, Muralidhar S (1998). Malignant syphilis: a review. AIDS Patient Care STDS.

[6] Vinay K, Kanwar AJ, Narang T, Saikia UN (2013). Malignant syphilis. Int J Infect Dis.

[7] Chen JQ, Cao YL, Man XY (2022). Malignant syphilis in a young woman: a case report. J Int Med Res.

[8] Montenegro-Idrogo JJ, Muñante R, López-Fuentes M, Sanz-Castro M, Ventura-León A, Chávez-Esparza G, García-Cortez Y (2023). Malignant syphilis as the presenting complaint of advanced HIV. Int J STD AIDS.

[9] Garbarino M C, Trila C, Heffner L (2020). Malignant syphilis in a patient with HIV. Medicina (B Aires).

[10] dos Santos TR, de Castro IJ, Dahia MM (2015). Malignant syphilis in an AIDS patient. Infection.

[11] Witkowski J-A, Parish L C (2022). The great imitator: malignant syphilis with hepatitis. Clinics Dermatol.

[12] Fustà-Novell X, Morgado-Carrasco D, Barreiro-Capurro A (2019). Syphilis maligna: a presentation to bear in mind. Actas Dermosifiliogr.

[13] Tosca A, Stavropoulos PG, Hatziolou E, Arvanitis A, Stavrianeas N, Hatzivassiliou M, Stratigos JD (1990). Malignant syphilis in HIV-infected patients. Int J Dermatol.

[14] Melian-Olivera A, Jimenez-Cauhe J, Moya-Martinez C (2023). Malignant syphilis. Indian J Dermatol Venereol Leprol.

[15] Narasimhan M, Lagoo M, Ramachandran R, Fernandes SD (2022). Syphilis D’ Emblée: a case series of the great masquerader. J Family Med Prim Care.

[16] Petersen E, Kantele A, Koopmans M, Asogun D, Yinka-Ogunleye A, Ihekweazu C, Zumla A (2019). Human monkeypox: epidemiologic and clinical characteristics, diagnosis, and prevention. Infect Dis Clin North Am.

